# Photovoltaic Materials and Their Path toward Cleaner Energy

**DOI:** 10.1002/gch2.202200146

**Published:** 2022-10-28

**Authors:** Aleksandar M. Mitrašinović, Milinko Radosavljević

**Affiliations:** ^1^ Institute of Technical Sciences of the Serbian Academy of Sciences and Arts Kneza Mihaila 35/IV Belgrade 11000 Serbia; ^2^ Mining Institute Batajnički put 2 Zemun 11080 Serbia

**Keywords:** clean energy, integrative technologies, materials, photovoltaics, solar cells

## Abstract

Photovoltaic silicon converts sunlight in 95% of the operational commercial solar cells and has the potential to become a leading material in harvesting energy from renewable sources, but silicon can hardly convert clean energy due to technologies required for its reduction from sand and further purification. The implementation of the novel materials into photovoltaic systems depends on their conversion efficiency limited by the material's inherent properties, longevity dependent on internal stability, and ease of manufacturing process. A major challenge is discovering a multilayered set of different photovoltaic materials capable of converting clean energy from a wider spectra range since emerging materials and technologies such as dye‐sensitized and quantum dots suffer from low conversion efficiencies while perovskite and organic cells have short longevity in atmospheric conditions. Presently, improving technologies for commercialized materials and creating multijunction solar cells enhanced by new photovoltaic materials is a path toward cleaner energies. With the rapid development of the integrative technologies and challenges that photovoltaics for clean energy conversion are facing, the entire clean photovoltaic industry could arise by bottom‐up course as a part of integrative technologies rather than erecting large power plants.

## Graphical abstract

Major determinators in distinguishing photovoltaic materials are conversion efficiency, energy payback, and cost‐per‐kilowatt. Nowadays, silicon is a leading material for renewable conversion of sunlight into electricity. The future clean photovoltaic conversion based on emerging materials may rise by bottom‐up principle as part of integrative technologies rather than the addition to the existing grid network or erecting large power plants.

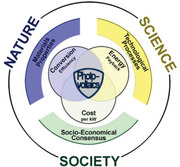

## 1 Introduction

**Figure 1:**
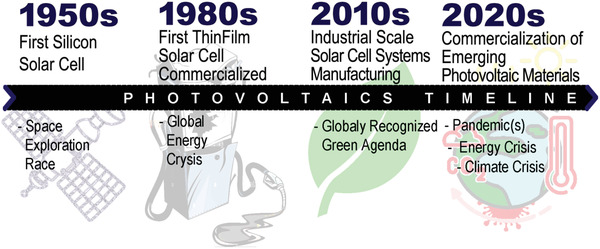
Progress of photovoltaics industry. Development of the photovoltaic industry proved tightly connected to the global socioeconomic fluctuations, where each energy crisis triggered a search for alternative sources of energy. The most notable interconnection between the global events and the subsequent development of the new photovoltaic materials and technologies are the space exploration race in the 1950s, the oil crisis in 1974, and the pursuit of a carbon‐free environment since the 2000s.

Major socioeconomic shifts on the global scale inevitably induce harsh periods for human societies, but these periods were traditional triggers for advancements in the photovoltaic sector (**Figure** **1**). During space explorations race in the 1950s, silicon solar cells from Bell Laboratories were the first photovoltaic systems used to convert photons’ energy into electricity.^[^ ^1^ ^]^ In the same decade, the silicon solar cells developed for space exploration were commercialized for terrestrial use. The global oil crisis in 1974 pushed research toward photovoltaic materials different from silicon resulting in the commercialization of the second‐generation thin‐film solar cells.^[^ ^2^, ^3^ ^]^ Increased awareness regarding the sustainability of our habitat and extensive use of fossil fuels at the beginning of the 21st century resulted in large governmental subsidies toward renewable energy sources.^[^ ^4^, ^5^, ^6^ ^]^ Consequently, the photovoltaic industry base shifted from small privately owned vendors to large companies with industrial size production and installation capacities.^[^ ^7^ ^]^ Simultaneously, the research regarding the photovoltaic phenomenon has moved from a few leading labs to virtually every lab around the world. In the early 2020s, we are in the middle of the pandemic, followed by damaged communication and supply lines, and at the beginning of the incoming energy crisis. These circumstances will inevitably lead to a higher share in energy consumption from already commercialized first and second‐generation solar cells, push further development of the new photovoltaic materials and technologies, and faster commercialization of the third‐generation solar cells.^[^ ^8^ ^]^

**Figure 2:**
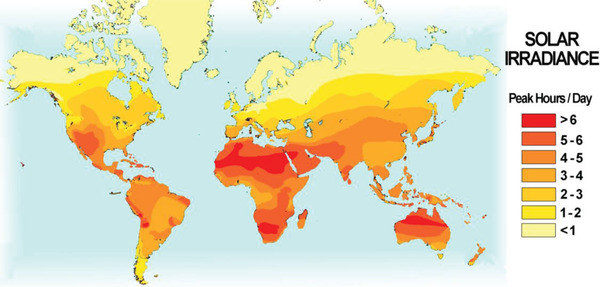
Terrestrial photovoltaic power potential. The majority of the human population lives in regions with at least three peak hours per day of solar irradiance that with current technological achievements allows one household to satisfy all its energy needs with a photovoltaic system. Data extrapolated from the World Bank Group website at globalsolaratlas.info.

The key advantage of photovoltaic technologies that makes them the most promising area of all renewable energy sources is that it does not pollute the environment in which operate, has no moving parts while having a lifespan of several decades with a low level of maintenance and monitoring. The photovoltaic technology does not require extremely high installation costs and it could be independent of the existing electrical network. Photovoltaic systems are easily installed in homes, public or commercial buildings, where their use is safe and silent. Remote and underdeveloped areas can easily produce their electricity by starting with a small energy system and further planning a gradual increase in capacity along with the increase in energy needs. Extremely low initial costs compared to erecting a traditional electricity grid make solar energy utilization the most preferred option in developing countries, where photovoltaic energy conversion is the fastest growing segment of the energy sector. Simultaneously, in some developed countries such as Canada, modular and decentralized photovoltaic technology is more effective than investing in long power transmission lines and is thus ideal for meeting the electricity needs of many remote households where the supply of electricity from the grid is unprofitable. For example, the most of Canadian Prairies lands are also one of the sunniest areas on the planet with 2350 h of direct sunlight a year,^[^ ^9^, ^10^ ^]^ which makes ideal conditions for solar cells: clear skies with low air temperatures that further increase the efficiency of solar cells.^[^ ^11^ ^]^ **Figure** **2** shows the number of peak sunlight hours on the global map and depicts the most suitable landmasses for solar systems installation. Solar cells also convert sunlight outside peak hours at a lower conversion rate, but the value of peak hours can be further increased by installing concentrators or sun‐directing mechanisms. Hence, in regions with just a few peak hours of direct sunshine, a household can satisfy its energy needs solely on photovoltaic systems. A typical home in North America uses 20 kWh per day, which corresponds to a 5 kW photovoltaic system in areas with only four peak hours per day. Typical silicon solar panels installed in earlier decades are rated at 250 W, which means a 5 kW system would require about 20 × 250 W solar panels at 1 × 1.6 m^2^ standard size while the above‐mentioned Canadian regions would need two‐thirds of that area. In 2021, premium commercially available systems provide guaranteed 350 W power with 30 years warranty that further reduces the required size of photovoltaic surface and longevity of installed photovoltaic systems.

The rapid development of new photovoltaic materials and technologies, and discoveries of the new material properties and conversion mechanisms require a constant update on inflowing data, where some widely accepted definitions and terminology are changing. The most commonly used classification of solar cells in the present time originated from the early commercial vendors of photovoltaic systems. Arranging photovoltaic materials into the first, second, and third generation solar systems refers to the sequence of introducing a particular technology to the global market. While the first and second generations of solar cells are matured regarding worldwide commercial distribution networks and are well investigated by researchers, most new photovoltaic materials and technologies introduced in the last two decades are classified into the third generation regardless of photovoltaic material, manufacturing technology, or conversion mechanism. Given many newly developed materials sensitive to incident light coming from the sun, this report emphasizes the need for narrowing and reiterating criteria and classification parameters regarding photovoltaic phenomena and related photovoltaic materials and technologies. Together with emphasizing the crucial parameters in distinguishing photovoltaic materials from a larger family of photosensitive materials (Sections 3 and 4), this report in Section 5 reveals current challenges in producing commercial photovoltaic materials and in Section 6 envisions further development of the materials and technologies for clean energy conversion.

## 2 Status of the Solar Energy Industry in the Early 2020s

**Figure 3:**
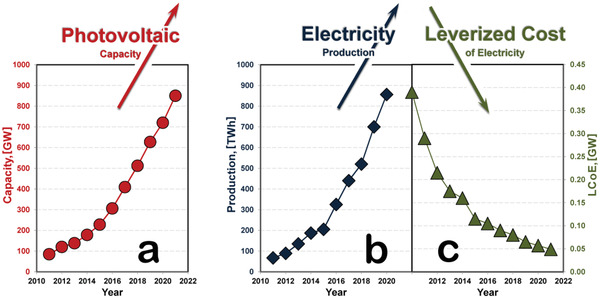
Photovoltaics statistics. An increase in global photovoltaic systems capacity achieved by gradual social acceptance since the 1070s and significant governmental subsidies since the 2000s a) led to an exponential increase in electricity production since the early 2010s b) with the significant decrease in Leveraged Cost of Electricity (LCoE) throughout 2010s c) that nowadays made photovoltaic industry independent, self‐sufficient, and profitable. Data extrapolated from International Energy Agency.^[^ ^22^ ^]^

In a few decades, photovoltaics‐based energy conversion technologies have evolved into a self‐sustained industry that experiences the highest growth in the renewable and green sector. Power generation from photovoltaic systems in 2020 is increased by 156 TWh that is 23% higher than in the preceding year.^[^ ^12^ ^]^ Electricity generated by solar systems accounted for 3.1% of global electricity generation positioning photovoltaics as the third‐largest renewable electricity technology behind hydropower and wind. Since silicon solar cells are present in the market for half of the century, ≈95% of the operational commercial solar cells in 2020 where based on silicon^[^ ^13^ ^]^ where the share of the higher quality mono‐crystalline technology raised to 84%, compared to 66% in 2019.^[^ ^14^ ^]^ The major producer of high purity silicon for the electronic industry remains to be Wacker Chemie aktiengesellschaft (Germany/USA), the same company that developed the Siemens process in the 1950s. Regarding the production of lower grade 6N solar poly silicon, China is a world leader by far in both annually installed capacity and overall production of the photovoltaic systems.^[^ ^15^, ^16^ ^]^ According to the International Renewable Energy Agency, China's installed solar cell system capacity was around 254 GW in 2020, about 50 GW higher than in 2019. These values will continue exponential growth as depicted in **Figure** **3** among various projects supported by China's Center for Renewable Energy Development^[^ ^17^ ^]^ the construction of the 400 GW solar and wind plants in desert areas started in 2021 with an expected commission in 2025^[^ ^18^ ^]^ and further lead toward China's promised carbon neutral target by 2060.^[^ ^19^, ^20^ ^]^ Further in the future, such developments may reach a plateau percent‐wise due to grown installed capacity but capacity wise these numbers will be noticeably higher each year.

The power output of the commercial multisilicon solar cells rose significantly from below 250 W 10 years ago to over 350 W nowadays. Development of the new photovoltaic materials is hindered in statistics‐based reports because about 95% of the commercial solar cells are based on silicon due to its long‐term presence in the market. However, the reports on emerging photovoltaic materials and technologies are stating the great increase in conversion efficiencies in the third‐generation solar cells. Regardless of the photovoltaic material or manufacturing technology, current innovations lead to increased solar systems’ overall capacity and power output without significantly increasing the manufacturing costs.^[^ ^21^ ^]^ Increased power for the equal amount of photovoltaic material further leads to a lower cost per kW value for new photovoltaic systems not only due to less used photovoltaic material but also other equipment (e.g., wires and junction boxes) used to convert energy inside photovoltaic material and to deliver this electricity to the grid.

## 3 Major Determiners in Photovoltaic Materials Considerations

With the ever‐growing number of new materials and compounds where many show sensitivity to incident sunlight, the distinction between photosensitive and photovoltaic materials becoming unclear, in particular in the early stages of research.^[^ ^23^ ^]^ Photovoltaic materials are traditionally defined by their unique ability to convert solar radiation into electricity. However, with the entering of, for example, quantum dot solar cells,^[^ ^24^ ^]^ photosynthesis emulative processes,^[^ ^25^ ^]^ or photocatalytic green hydrogen production^[^ ^26^ ^]^ where energy is used for chemical reactions, the traditional definition of photovoltaic materials is becoming doubtful. Some materials can exhibit sensitivity to solar radiation but cannot sustainably generate electricity. For example, a vast number of inorganic compounds are photosensitive but hardly all of them can be used as sustainable photovoltaic material.^[^ ^27^ ^]^ Nevertheless, as some material converts sunlight to electricity, the value of generated power should exceed the amount of energy invested in extracting or synthesizing the photovoltaic material, and degradation of the conversion efficiency throughout service time should be prolonged enough to satisfy efficacy standards. Phenomena such as upconversion can be used for sensors while downconversion is commercialized in light emitting diode lighting, but both processes require more energy that can bring back to the electrical circuit. **Table** **1** shows the required determination for one substance to be considered a photovoltaic material. Conversion efficiency and energy payback time depend on materials properties and determine the ability of the particular material to generate power from the photons. Cost per kW and market trend determine the commercialization potential of the photovoltaic materials. Along with modern societies moving toward carbon neutral economies, CO_2_ payback time will become another unavoidable determinator in photovoltaic materials considerations.

**Table 1:** Required determinators in photovoltaic materials considerations

Determinator	Parameter	Description
Conversion efficiency	Physical boundary	Does the maximum power that one material can provide justifies further considerations?
Energy payback	Feasibility boundary	Does the total energy invested in one material exceed the maximum generated power over the entire life cycle?
Cost per kW	Human motivator	Will the money invested generate profit?

**Figure 4:**
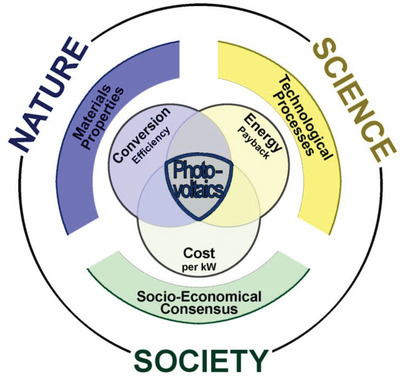
Photovoltaics’ entanglement between nature, science, and society. Major determinators in photovoltaic materials considerations are governed by inherited material properties set by nature, human knowledge regarding technologies available for generating photovoltaic systems, and overall acceptance of a particular society to move forward from the dependence on carbon‐based energy sources.

With each innovation in design and technology, newer types of photovoltaic materials improve characteristics and more controllable synthesis procedures. Regardless of the material, technology, or conversion mechanism, each photovoltaic cell has to satisfy key properties that characterize solar cell systems (**Figure** **4**). The conversion efficiency of the solar cell has to be high enough to justify the cost of further R&D, installation, and maintenance costs over the service time. Some materials demonstrate excellent conversion efficiency values, but energy invested in their extraction from the Earth's crust, purification, and installation can exceed acquired energy from the photovoltaic process over an extended period. Energy payback time provides a ratio between converted energy during solar systems’ service time and energy invested into the same solar system. Moreover, energy payback time values should be much lower than the photovoltaics’ lifetime to be considered for further commercialization. Among the two the most important factors that determine photovoltaic material sustainability and further economical validation, conversion efficiency relates to the physical properties of the photovoltaic material, while energy payback time relates to technologies for particular photovoltaic material production and installation.

## 4 Conversion Efficiency, Internal Stability, and Manufacturing Processes of the Photovoltaic Materials

The eventual implementation of the novel material into commercialized photovoltaic systems mainly depends on their conversion efficiency limited by the inherited properties of the photovoltaic component defined by Shockley–Queisser limit, longevity dependent on internal stability, and ease of the manufacturing process. The materials with photovoltaic characteristics are often classified based on the period when particular material and technology become commercial. The current market is almost exclusively covered by the first and second solar cell generations. First‐generation silicon and gallium‐arsenide solar cell systems characterize moderate conversion efficiencies but excellent structural and internal stability and well‐known production technologies. The thin‐film technologies are often referred to as the second‐generation solar cells that have lower conversion efficiencies compensated by low manufacturing cost and ease of installation on various surfaces. The recently developed technologies and novel photovoltaic materials are coming in a great variability of designs, materials, and manufacturing methods that have great potential regarding theoretical conversion efficiencies, low manufacturing costs, scalability, flexibility, etc. However, a common challenging factor for all novel photovoltaic systems is its steadiness and strength over a long time, leading to the conclusion that the leading role of silicon as a major commercial photovoltaic material will finish once new material and technology achieve at least internal stability and longevity of the silicon solar cells systems.

### 4.1 Conversion Efficiency

**Figure 5:**
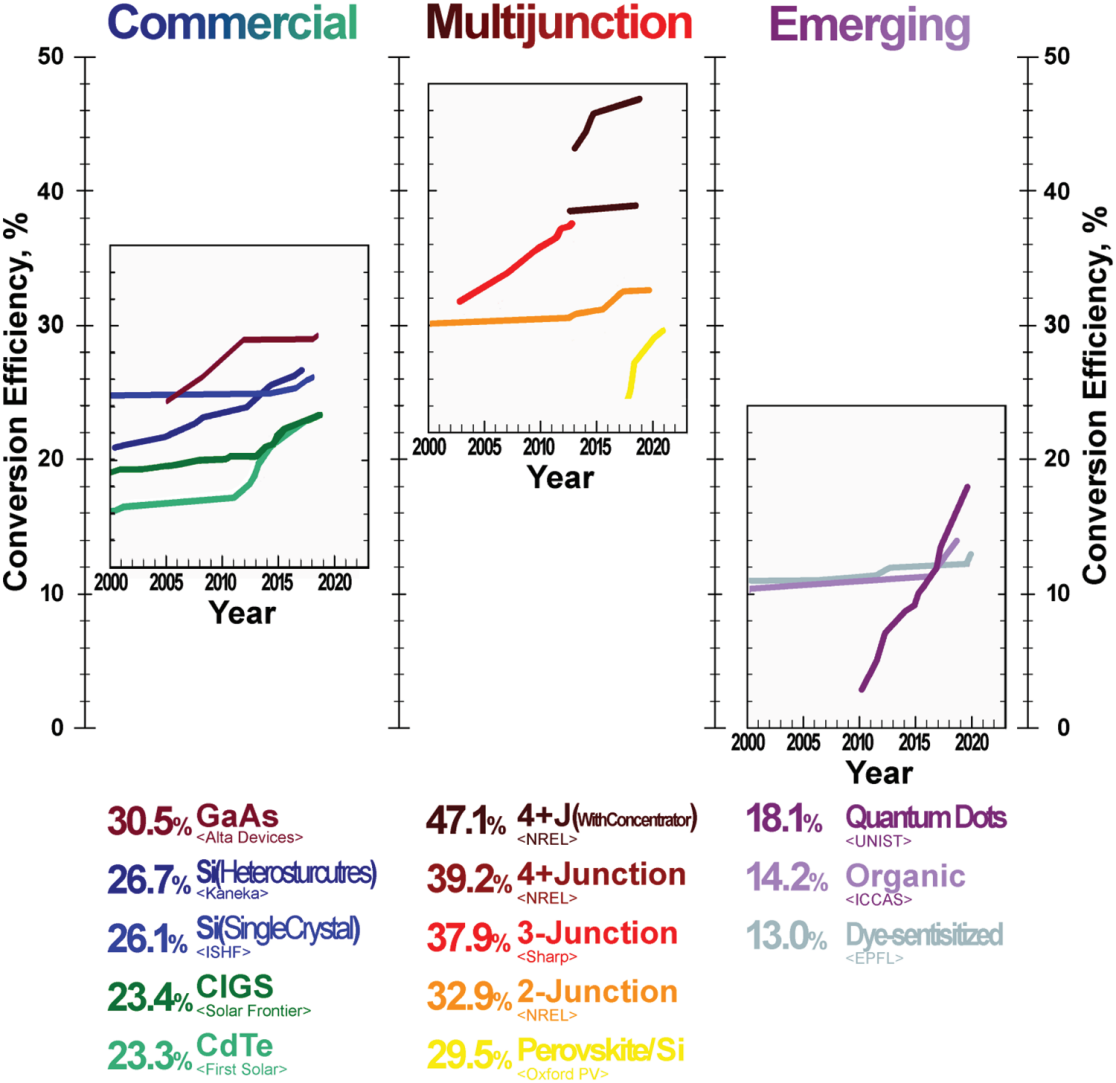
Conversion Efficiencies. An increase in the highest conversion efficiencies in the 21st century of the photovoltaic systems based on different materials reported by research labs or solar cells suppliers^[^ ^31^, ^32^ ^]^ shows that multijunction solar cells by utilizing different photovoltaic materials offer a unique advantage of converting photon's energy from a wider spectra range, while emerging photovoltaic materials jet suffers from significantly lower conversion efficiencies but even more important lower internal stabilities and imperviousness to atmospheric conditions.

The solar spectrum has a wide range, from 100 to 10 000 nm where the visible part of the spectrum is between 400 and 700 nm. Considering the large spectra of the solar spectrum, numerous materials absorb this energy and produce different phenomena rather than the photovoltaic effect. The different photosensitive materials convert electromagnetic energy only in a specific spectral range. Hence, a rather large part of the incident sunbeam would not excite electrons because some photons do not have enough energy, e.g., photons below 1.1 eV in silicon‐based solar cells while photons with surplus energy transform their surplus into heat that reduces the overall efficiency of the solar cells. Each photovoltaic material has a unique theoretical maximum limit of conversion efficiency, i.e., nearly 29% for mono‐crystalline silicon. Besides inherited losses regarding bandgap value for particular photovoltaic material, there are various losses related to the entire system responsible for converting the sun's energy to electricity, e.g., optical losses, reflections of incoming rays, electrical resistance losses, material contamination, surface and crystal defects, etc. Researchers from the National Renewable Energy Laboratory (NREL) demonstrated the world's highest 47.1% conversion efficiency solar cell operated under the direct spectrum at 143 Suns concentration, using a monolithic‐series‐connected six‐junction inverted metamorphic structure.^[^ ^28^ ^]^ Far beyond multijunction solar cells, perovskite/Si tandem solar cells reached 29.5% conversion efficiency^[^ ^29^ ^]^ while other emerging photovoltaic materials, e.g., organic or quantum dots cells, have conversion efficiencies below 20%.^[^ ^30^ ^]^ Among silicon and thin‐film solar cells, heterostructures silicon and copper indium gallium selenide (CIGS) have the highest conversion efficiencies at 27.6% and 23.4%, respectively. **Figure** **5** maps the highest reported conversion efficiencies for various photovoltaic systems over the time that visualize circumstance where particular value for the efficiency of the solar cells occurred with its discovery does not change significantly later were these incremental improvements were associated with new synthesis and assembly methods rather than changes in photovoltaic material itself.

### 4.2 Internal Stability

The conversion efficiency data alone does not give information on how long solar cells provide maximum output and how long these solar cells can keep photovoltaic properties after its syntheses.^[^ ^33^ ^]^ In first‐generation solar cells, the structural and internal stability of silicon solar cells were rarely discussed. Some solar cells of the first generation are still operational, 40 years after its installments. One of the major drawbacks of third‐generation solar cells is their internal stability over a long time. While some scientists claim that there are no insurmountable barriers regarding the internal stability of the solar cells, each material with photovoltaic capabilities does have its unique inherited limitations that are difficult to mitigate.^[^ ^34^ ^]^ For example, in the first two decades of the 21st century, a technological breakthrough is made in synthesizing organic or hybrid photovoltaic research but despite their low production cost and low environmental impact, their market share remains small due to the vulnerability of the organic components on atmospheric or bio microbial impacts. Some concept solar cells like black silicon imply structural stability similar to the first generation solar cells why some quantum dots materials preserve internal stability by being encapsulated inside a transparent body. However, uncertainties regarding molecular stability on temperature increase in organic and some hybrid solar cells, as well as the toxicity of some components, are yet to be answered on the research level before entering the development and eventually commercialization phase.

The degradation rate of the silicon and thin‐film solar cells is widely available in literature since their commercial use started five decades ago. Most photovoltaic‐module manufacturers offer a 25‐year or 30‐year warranty that corresponds to a power drop of less than 20% within the warranty period or ≈0.6–0.7% a year. According to an NREL report^[^ ^35^ ^]^ solar cells manufactured after 2000 have degradations rates 0.4%, 0.6%, 1.0%, and 0.5%, for mono‐Si, poly‐Si, a‐Si, and CdTe solar cells, respectively. Currently, First Solar from California claims the lowest degradation rate in the market.^[^ ^36^ ^]^ Series 6 CuRe has a 0.2% degradation rate during a 30‐year warranty achieved by replacing copper with elements from the fifth column of the periodic table (arsenic, antimony, or bismuth). An indicative observation is that degradation in first and second solar cell systems is below one percent per year and is mainly attributed to the solar cell parts rather than to the photovoltaic material itself. In opposite, third‐generation solar cells suffer from two digits degradation rates mostly attributed to the internal stability of the photovoltaic materials. Photovoltaics based on organic materials, even on a laboratory scale, are hardly achieving efficiencies above 10% while organic compounds easily decompose under incident light often reducing the lifespan of panels to months or weeks instead of years.

### 4.3 Manufacturing Processes

Advancements in manufacturing processes led to the specifications of the commercial solar systems being close to calculated Shockley–Queisser limits of the particular photovoltaic material.^[^ ^37^, ^38^ ^]^ Nowadays, major suppliers of silicon solar panels guarantee efficiencies above 24% with 30 years of warranties that are very close to the best laboratory results. Second the best commercial solar cell LG NeonR N‐Type solar modules have guaranteed efficiency of 22% at 380 W maximum power that is double the capacity of what was available in the early 2010s. Such rapid advances in solar cell efficiencies were not forecasted even by the most respected experts and government agencies, e.g., Japanese PV2030 road map from 2006 setting required efficiencies of 22% should be reached by the year 2030 for module and the wafer thickness to 50–100 µm.^[^ ^39^ ^]^ However, these advances were not achieved by discoveries and implementations of the new technologies but rather by small step improvements in every step in the rather long process of producing silicon solar cells from sand.^[^ ^40^ ^]^

**Figure 6:**
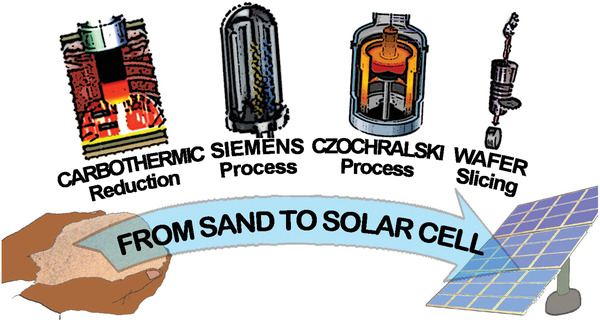
From sand to a solar cell. Manufacturing path of the most dominated material for photovoltaic conversion, monocrystalline silicon.

The vast majority of currently made commercial solar cells are designed to convert solar energy by using silicon as the main photovoltaic material. Almost the entire commercial production of feedstock silicon with required photovoltaic characteristics is based on the same process steps with very little processing or technological variations (**Figure** **6**). Metallurgical grade silicon with a purity far beyond photovoltaic requirements is produced from sand by a carbothermic reduction in electric arc furnaces. To produce cleaner silicon, extremely corrosive chlorine‐based gases are required where silicon first reacts with these gases and forms gaseous compounds that later on are distilled and flashed over the pure silicon rod to deposit silicon from gaseous compounds. Purification of silicon generated by the carbothermic process to extremely pure silicon by using chlorine‐based gases is known as the Siemens process. Siemens process provided the first batches of pure silicon for the electronic and photovoltaic industry in the 1950s and ever since remains the main process for industrial‐scale production of the feedstock silicon for the electronic and photovoltaic industry. Since conversion efficiency of the silicon solar modules is significantly higher for single crystal solar cells, the majority of purified silicon is melted and grown into large cylindrical single crystal ingots by the Czochralski method for directional solidification. Slicing is the final step in the treatment of the silicon feedstock material that creates wafers ready for processing in the functional solar cell. Although the final product, photovoltaic silicon, gives us renewable energy, all processes for producing that silicon are environmentally intensive.

## 5 Environmental Considerations Regarding Photovoltaic Materials for Clean Energy

Historically, new technologies were acquired and developed based on profitability estimates. Nowadays, societies with established environmental concerns related to environmental impact, sustainability, reusability, or recyclability would prevail toward environmentally friendlier technologies instead of pure profitability assessment. Photovoltaic systems are produced from a variety of technologies that have an impact on the environment. The most commonly used photovoltaic material silicon is produced from quartz by a carbothermic process that consumes a lot of energy and emits a significant amount of carbon dioxide from sources like coal or carbonaceous fossil fuel. Sice et al.^[^ ^41^ ^]^ estimated the life‐cycle energy requirement for crystalline silicon between 2700 and 5200 MJ m^−2^ and greenhouse emission rate from 23 to 45 g CO_2_‐eq kWh^−1^. Further refining up to solar grade quality requires even more corrosive gases such as halogenic silanes. Regardless, those productions of solar panels become highly automated processes contributing to a decrease in cost per kW value but automated processes are also consuming energy. Aluminum frames, ethylene vinyl acetate, wires, and glass sheet are important components of solar panels that require energy for their production. Apart from manufacturing the components, energy is expended for obtaining raw materials, processing, testing, packing, transportation, and installation of solar panels, where each step has an impact on the environment. The photovoltaic industry developed relatively recently and recycling solar panels at the end of their service time will become another environmentally challenging task.^[^ ^42^ ^]^ Revealed by recent global geo surveillance, the global inventory of commercial, industrial, and utility‐scale photovoltaic (PV) installations overlay the most valuable arid lands,^[^ ^43^ ^]^ which requires changes in policymaking toward installations in remote areas unaffected by human and biodiversity activities.

The rigid policy adjustments in developed countries regarding carbon emissions further increased interest for emerging photovoltaic materials since parameters aiming at environmental protection such as Life‐Cycle‐Assessment and CO_2_‐equivalent are becoming major determinator in photovoltaic considerations. In perspective, CO_2_‐eq kWh^−1^ will replace cost‐per‐watt parameter as key human motivator (see Table 1). Increased awareness regarding our environment and push toward carbon neutral societies also significantly improved both direct and indirect emissions related to energy input in photovoltaic silicon production.^[^ ^44^ ^]^ In 2011, Rich et al.^[^ ^45^ ^]^ found that heat supply mainly provided by the combustion of natural gas or oil contributes by 16 g CO_2_‐eq kWh^−1^; Supposing coal is used than CO_2_‐eq kWh^−1^ would rise to 30–200 g whereas considering energy used by a photovoltaic system by itself or other renewable resource would virtually eliminate emissions entirely. CO_2_‐equivalent for the emerging photovoltaic materials is difficult to set because their early research stage, but some techniques have potential to convert sunlight while produced with zero environmental impact.

## 6 Development Path toward Cleaner Photovoltaic Materials

Throughout the 2010s, it has been thought that either monocrystalline or polycrystalline silicon solar cells would be made obsolete by the eventual introduction of very inexpensive alternative solar cells based on thin films of semiconductors such as cadmium telluride (CdTe), copper–indium diselenide, or organic photovoltaic materials. This may happen, but so far, based on reliability, performance, and lifetime, none of these alternatives appear to be in a position to challenge silicon solar cells.^[^ ^46^ ^]^ Occurrences in the 2010s indicate an even stronger silicon position in the photovoltaic market, despite a strong marketing approach and commercialization of newly developed photovoltaic materials. Fortuitously, with recent refocusing on clean energies, interest in known competitor materials for silicon will decrease while the installed capacity of the silicon‐based solar cells will increase as long as new materials and technology for environmentally sustainable processes are discovered. These facts are mostly related to the silicon for renewable energies but photovoltaic materials capable of replacing silicon without leaving environmental traces are on the long way toward commercialization. **Table** **2** summarizes the path of the major photovoltaic materials regarding corresponding production technology, market share, and environmental impact in our common pursuit of clean energy.

**Table 2:** Current classification of the photovoltaic materials with most notable examples

	Solar cells				
Source of energy	Renewable				Clean
Generation	First	Second	Third		
Technology	Wafer	Thin film	Enabling	Integrated/Concept	Unknown
Market share	Major	Specialized	Emerging	Innovative	–
Environmental impact	High	High/moderate	n/a	n/a	None
Photovoltaic material	Mono‐Si Poly‐Si GaAs	a‐Si CdTe CIGS	Perovskite Quantum dots Dye‐sensitized	Organics Photosynthesis emulative	Uncertain

If the photovoltaic industry aiming to become the primary source of energy, that means the size of today's photovoltaic systems requires a tremendous capacity increase in a brief time.^[^ ^47^ ^]^ Along with environmental sustainability, the key advantage of the emerging photovoltaic materials should be its scalability. First generation GaAs PV cells would require 40 years of today's gallium production capacity to reach one terawatt (TW) electricity generation while second generation thin‐film CdTe modules would require 80 years of today's tellurium, and thin‐film CIGS would require 32 years to achieve the same goal.^[^ ^48^ ^]^ Likewise, it would only take a few days to scale up perovskites’ production capacity to the one TW level^[^ ^49^ ^]^ while some emerging photovoltaic materials would require even less time. The only commercial photovoltaic material that is scalable to this level is silicon, which for one TW electricity generation capacity would require around 80 d of our current silicon production.^[^ ^50^ ^]^

## 7 Perspective

The European Union in its European Green Deal set the goal of reducing 55% of its greenhouse gas emissions by 2030 and becoming the completely climate neutral by 2050^[^ ^51^ ^]^ where increasing renewable energy share to 38.5% is essential to achieve the agreed objectives.^[^ ^52^ ^]^ Increasing the market share of new photovoltaic materials inevitably leads to further improvement in the photovoltaic field. Considering increased awareness regarding our natural habitat and global consensus regarding replacing fossil fuels with renewable sources of energy, further advancement will continue. Most strategic plans rely on incorporation of the renewable sources of the energy into the existing high power density systems. Inherited lack of long‐term stability and rapid degradation in atmospheric conditions are unsolvable drawbacks for emerging photovoltaic materials in competition with not only fossil fuels but with first and second generation of the photovoltaic materials. However, emerging photovoltaic materials become preferable materials in net‐zero energy buildings, transportation vehicles, agri‐lands, specialized habitats or entire human habitation systems. The environmental and economic evaluation of the household energy management connected to high power density systems and the solar system always gives the same outcome; grid connected is significantly lower‐cost while solar systems are solutions are less impactful on environment.^[^ ^53^ ^]^ Integrating emerging photovoltaic materials into urban environment where buildings infrastructure provides better protection from atmospheric influences and better cooling may mitigate some of its key hindrances of the emerging photovoltaic materials.

Considering cleaner methods for energy conversion, photovoltaics become a key component of the relatively new scientific discipline: integrative technologies. Wu et al.^[^ ^54^ ^]^ recently stated that flexible perovskite/c‐Si tandem technology with excellent 27.6% conversion efficiency is the most competitive candidate for the next generation mass‐produced solar cells integrated on the buildings’ exterior while Roger et al.^[^ ^55^ ^]^ suggested technological solutions for commercially viable production of similar material. Simultaneously, flexible photovoltaic materials capable of high‐power conversion efficiency in the low light environments become developed for indoor applications^[^ ^56^ ^]^ while near‐infrared dye‐sensitized solar cells can satisfy high aesthetic requirements, if required.^[^ ^57^ ^]^ Researchers from the German Aerospace Center^[^ ^58^ ^]^ gave a substantial report regarding adopting solar cells into versatile applications, including buildings, vehicles, extraterrestrial applications, etc.

In a view of the integrative technologies, it may happen that the entire clean photovoltaic industry may naturally rise by bottom‐up principle as part of integrative technologies rather than the traditional top‐down approach by extensively planning, financing and finally erecting large power plants on valuable landfills. In the next decade, we will witness total energy consumption significantly deviates from the consumption from the grid networks that would indicate a significant intake of the emerging photovoltaic technologies integrated with buildings, agricultural fields, vehicles, road infrastructure, etc. Further integration of photovoltaic materials into human habitats will continuously decrease an environmental impact of these materials until finally new net‐zero energy technologies with zero values for CO_2_ equivalent emission become available.

## 8 Conclusions

In a shift toward clean energies, photovoltaic technologies are the most promising solution to the global ever‐growing energy demand. A significant number of new photovoltaic materials will find their market. Commercialized in the previous century, photovoltaic materials used in the first and second‐generation solar cells reached their inherited conversion efficiency limits and their further improvements rely on advancements in photovoltaic systems rather than on the material itself. If only conversion efficiency is considered, some third‐generation multijunction cells introduced in the market achieved energy conversion rates above 40%.^[^ ^59^ ^]^ A variety of the materials and technologies implemented in third‐generation solar cells indicate even higher conversion efficiencies in a laboratory environment, but the internal stability of the photovoltaic materials limits their implementation on an industrial scale. Major determinators in distinguishing photovoltaic materials from the larger family of photosensitive materials are conversion efficiency, energy payback, and cost‐per‐kilowatt. Further assessment regarding the commercialization of the photovoltaic materials relies on the internal stability of the photovoltaic material, manufacturing technology, and environmental impact of the process. In the meantime, before photovoltaic materials for sustainable clean energy are found, paradoxically, photovoltaic silicon that is produced by environmentally intensive processes will remain the leading material for converting sunlight into electricity. With the rapid development of the integrative technologies and challenges that photovoltaics for clean energy conversion are facing, it would not be a surprise if the entire clean photovoltaic industry arises as a part of integrative technologies rather than building large power plants as part of the existing network.

## Conflict of Interest

The authors declare no conflict of interest.
